# Aucubin Protects against Myocardial Infarction-Induced Cardiac Remodeling via nNOS/NO-Regulated Oxidative Stress

**DOI:** 10.1155/2018/4327901

**Published:** 2018-06-25

**Authors:** Zheng Yang, Qing-Qing Wu, Yang Xiao, Ming Xia Duan, Chen Liu, Yuan Yuan, Yan-Yan Meng, Hai Han Liao, Qi-Zhu Tang

**Affiliations:** ^1^Department of Cardiology, Renmin Hospital of Wuhan University, Wuhan 430060, China; ^2^Cardiovascular Research Institute, Wuhan University, Wuhan 430060, China; ^3^Hubei Key Laboratory of Cardiology, Wuhan 430060, China

## Abstract

Whether aucubin could protect myocardial infarction- (MI-) induced cardiac remodeling is not clear. In this study, in a mouse model, cardiac remodeling was induced by left anterior descending coronary artery ligation surgery. Mice were intraperitoneally injected with aucubin (10 mg/kg) 3 days post-MI. Two weeks post-MI, mice in the aucubin treatment group showed decreased mortality, decreased infarct size, and improved cardiac function. Aucubin also decreased cardiac remodeling post-MI. Consistently, aucubin protected cardiomyocytes against hypoxic injury in vitro. Mechanistically, we found that aucubin inhibited the ASK1/JNK signaling. These effects were abolished by the JNK activator. Moreover, we found that the oxidative stress was attenuated in both in vivo aucubin-treated mice heart and in vitro-treated cardiomyocytes, which caused decreased thioredoxin (Trx) consumption, leading to ASK1 forming the inactive complex with Trx. Aucubin increased nNOS-derived NO production in vivo and vitro. The protective effects of aucubin were reversed by the NOS inhibitors L-NAME and L-VINO in vitro. Furthermore, nNOS knockout mice also reversed the protective effects of aucubin on cardiac remodeling. Taken together, aucubin protects against cardiac remodeling post-MI through activation of the nNOS/NO pathway, which subsequently attenuates the ROS production, increases Trx preservation, and leads to inhibition of the ASK1/JNK pathway.

## 1. Introduction

Adverse left ventricular (LV) remodeling includes complex changes in LV size, morphology, function, and cellular molecules [1]. Inflammation, apoptosis, fibrosis, and the maturation of collagen scar remodel the heart after MI [1, 2]. Although early inflammation, apoptosis, and fibrosis are necessary events for cardiac repair, disproportionately prolonged inflammation, excessive apoptosis, and overactive fibrotic responses can lead to sustained tissue damage and increased cell loss and improper healing, thus promoting expansion of the infarct area and leading to chamber dilatation [3, 4]. In clinical practice, therapeutic manipulation of the ensuing repair process has proved much more challenging and elusive. Therefore, exploring new therapeutic strategies that effectively target this detrimental process is of great importance.

Aucubin is a natural compound that can be extracted from the leaves of *Aucuba japonica* and *Eucommia ulmoides* [5]. It shows multiple pharmacological effects, including anti-inflammatory [6, 7], antiapoptosis [8, 9], neuroprotective [10], and antioxidative [5, 11] properties. By inhibition of NF-*κ*B signaling, aucubin prevents proinflammatory cytokine-induced inflammatory response [6, 7]. Aucubin is also reported to regulate Bcl-2 family protein expression and inhibit cell death [12] and apoptosis [8, 9]. Ho et al. reported that aucubin protected against UVB-induced skin fibroblast activation by inhibition of the production of matrix metalloproteinase-1 [13]. Since inflammation, cardiomyocyte apoptosis, and fibrosis are all involved in the progression of cardiac remodeling after MI [1], aucubin was supposed to have protective effects on cardiac remodeling post-MI because of the anti-inflammatory, antiapoptotic, and antifibrotic properties. Until now, it has remained unclear whether aucubin could retard or even reverse cardiac remodeling post-MI. Hence, the aim of our study was to investigate the protective effect of aucubin on LV remodeling after MI.

## 2. Materials and Methods

### 2.1. Reagents

Aucubin (98% purity) was purchased from Shanghai Winherb Medical S&T Development Co. Ltd. (Shanghai, China). The primary antibodies against Bax, Bcl-2, c-caspase 3, TNF*α*, phosphorylated and total JNK, nNOS, and GAPDH were purchased from Cell Signaling Technology (MA, USA). The antibodies against total ASK1, SOD, Trx, gp91, P67, iNOS, and eNOS were purchased from Abcam (Cambridge, UK). The antibodies against phosphorylated ASK1 were purchased from Santa Cruz Inc. (TX, USA).

### 2.2. Animals

All animal procedures were performed in accordance with the Guide for the Care and Use of Laboratory Animals published by the US National Institutes of Health (NIH Publication Number 85-23, revised 1996) and approved by the Animal Care and Use Committee of Renmin Hospital of Wuhan University. Eight- to ten-week-old male C57/BL6 mice were purchased from the Institute of Laboratory Animal Science (Beijing, China). nNOS-KO mice were purchased from Jackson Laboratory. The animals were randomly assigned to 4 groups: vehicle-sham group, aucubin-sham group, vehicle-MI group, and aucubin-MI group. The administration of aucubin (10 mg/kg, intraperitoneal injection) was performed 3 days after MI surgery and maintained for a further 11 days.

### 2.3. Left Coronary Artery Ligation Surgery

The left coronary artery ligation (LAD) surgery was performed, as in our previous study [14]. Briefly, mice were anesthetized by sodium pentobarbital (50 mg/kg, ip). After opening the pericardium, a 7-0 silk suture was used to ligate the left coronary descending artery. In sham-operated mice, the left coronary artery was encircled without ligation in sham surgery mice. The operations and all analyses were performed blinded.

### 2.4. Echocardiography and Hemodynamics

Echocardiography and hemodynamic measurement were performed, as described in our previous study [14]. Briefly, a MyLab 30CV ultrasound (Biosound Esaote) was used.

For hemodynamic measurement, a microtip catheter transducer (SPR-839; Millar Instruments, Houston, TX) was used.

### 2.5. Histological Analysis and Immunohistochemistry

Hematoxylin and eosin (HE) staining and Masson trichrome staining were performed, as our previous study described [14]. For immunohistochemistry, the heart sections were incubated with primary antibodies anti-CD68 (ABD Serotec, MCA1957) and anti-CD45 (Abcam, ab10558). Then, sections were incubated with EnVision™+/HRP reagent and stained with a DAB detection kit.

### 2.6. TUNEL Staining

TUNEL staining was performed, as our previous study described [14]. Briefly, a TUNEL assay (Millipore, USA) was used according to the manufacturer's instructions. A fluorescence microscope (Olympus DX51) was used to evaluate apoptotic cells.

### 2.7. NO and ROS Detection

NO production was determined as the measurement of nitrate plus nitrite using the Griess reaction assay (Cayman Chemical, Ann Arbor, MI) according to the manufacturer's instructions [15].

A 2,7-dichlorodihydrofluorescein diacetate (DCF-DA, Invitrogen) was used to detect ROS level with a microplate reader to detect excitation wavelength of 488 nm and emission at 525 nm.

### 2.8. RT-PCR and Western Blot Analysis

RT-PCR and Western blot were performed, as our previous study described [14]. Briefly, total RNA was extracted and reverse-transcribed into cDNA. A LightCycler 480 SYBR Green 1 Master Mix (04707516001; Roche) was used to quantify amplification. The GAPDH gene was used as reference.

For Western blot, cardiac tissue and cardiomyocytes were lysed and then loaded on an SDS-PAGE. After transfer to a membrane, primary antibodies were used to incubate the blot. After incubation with secondary antibodies, the blots were scanned by a two-color infrared imaging system (Odyssey, LI-COR). GAPDH protein was used as the reference.

### 2.9. Cell Culture

The H9c2 cell culture was performed, as our previous study described [14, 16]. Cells were pretreated with aucubin (1 *μ*M, 10 *μ*M, and 50 *μ*M) and/or NAC (10 mM), L-NAME (100 *μ*M), L-VINO (10 *μ*M), and L-canavanine (1 mM) for 12 h and then exposed to hypoxia in a BioSpherix C-Chamber (5% oxygen) for 24 h. Cells in the control group were cultured in a 5% CO_2_ and 95% air at 37°C.

### 2.10. Statistical Analysis

The data are presented as the mean ± SE. A one-way analysis of variance followed by Tukey's post hoc test was used to analyze differences among groups. Student's *t*-test was used to analyze differences between two groups. A *p* value less than 0.05 was considered significant.

## 3. Results

### 3.1. Aucubin Improves Survival Rates and Postinfarction Cardiac Function

**Figure 1:**
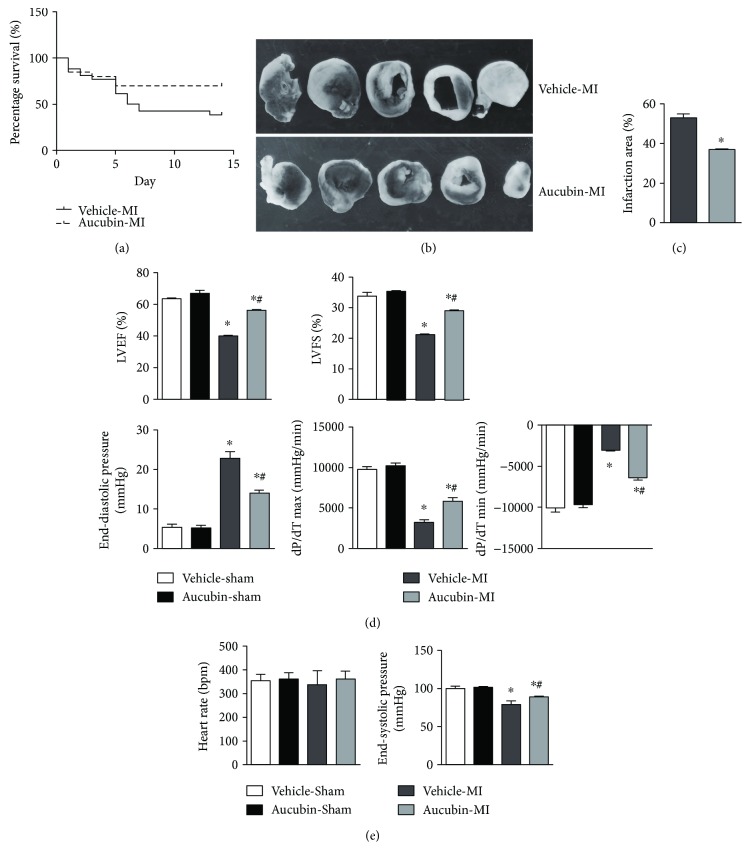
Aucubin improves survival rates and postinfarction cardiac function. (a) Kaplan-Meier survival analysis of mice in the vehicle-MI and aucubin-MI groups in the first 2 weeks after MI. (b) and (c) Triphenyltetrazolium chloride (TTC, 1%, Sigma, USA) staining of mouse hearts in the vehicle-MI and aucubin-MI groups 2 weeks after MI ((b) representative image and (c) quantitation result). ^∗^*p* < 0.05 versus vehicle-MI. (d, e) Echocardiographic ((d) LVEF, LEFS) and hemodynamic ((e) heart rate, ESP, EDP, dP/dtmax, and dP/dtmin) results for mice in the four groups at 2 weeks post-MI (*n* = 6–8). ^∗^*p* < 0.05 versus vehicle-sham and ^#^*p* < 0.05 versus vehicle-MI.

Mice in the sham group were alive at the end of the observation period in both the aucubin and vehicle-treated sham groups. The survival rate of the mice in the vehicle-MI group was significantly lower than in the aucubin-MI group (61.6% versus 30%, respectively; *P* < 0.05; Figure 1(a)). In addition, triphenyltetrazolium chloride (TTC, 1%, Sigma, USA) staining showed that aucubin reduced the ratio of infarct size at 2 weeks after MI, as shown in Figures 1(b) and 1(c).

Echocardiography and hemodynamic measurements revealed that aucubin improved cardiac dysfunction 2 weeks post-MI, as evidenced by increased LVEF, LVFS, dP/dtmax, dP/dtmin, and ESP and decreased EDP in aucubin-treated mice compared with that in vehicle-treated mice (Figures 1(d) and 1(e)). However, aucubin did not affect the heart rate in either the sham or MI groups (Figure 1(e)). It is worth noting that under basal conditions, aucubin administration did not affect the normal cardiac structure or function.

### 3.2. Aucubin Attenuated Cardiac Hypertrophy and Fibrosis Post-MI

**Figure 2:**
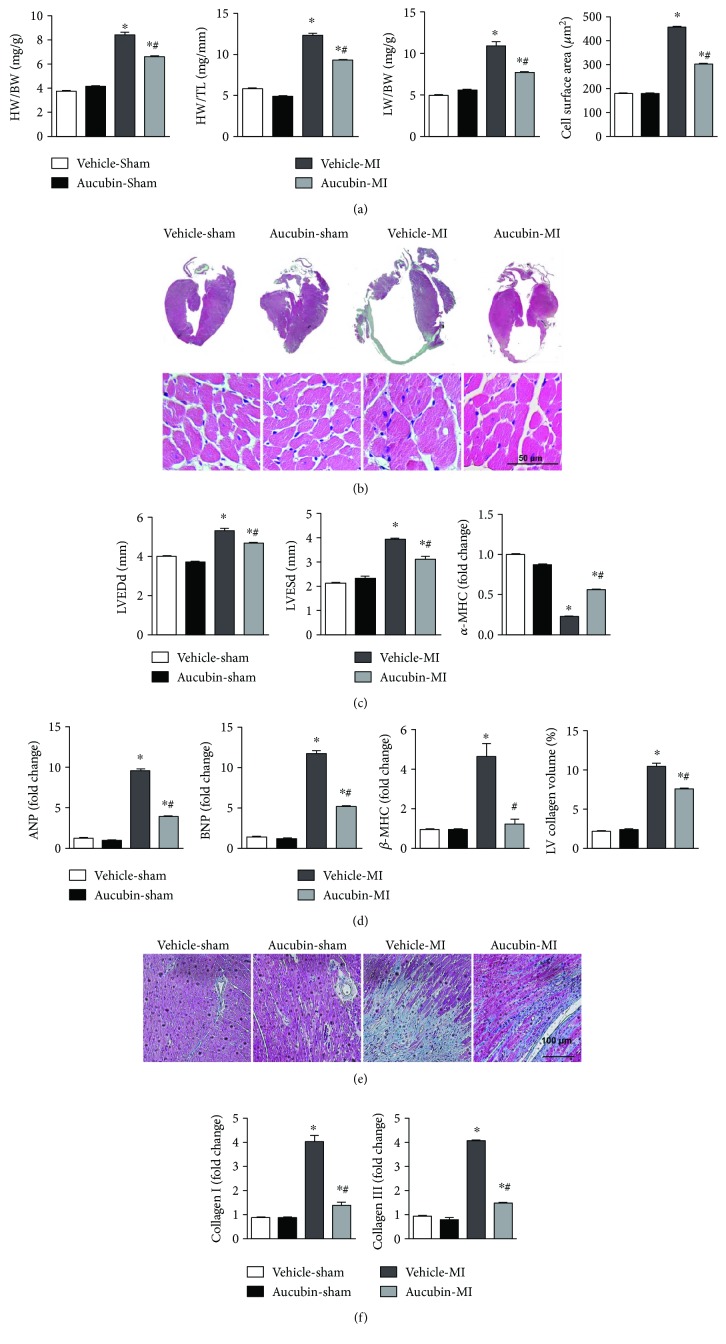
Aucubin attenuated cardiac hypertrophy and fibrosis post-MI. (a) Statistical analysis of heart weight (HW)/body weight (BW), HW/tibial length (TL), and lung weight (LW)/BW in the mice from indicated groups post-MI (*n* = 6–8). (b) H&E staining (left, *n* = 6) and statistical analysis of the cross-sectional area (right, CSA, *n* = 100+ cells per experimental group). (c) The echocardiographic measurement of LVEDd and LVESd levels in the indicated mouse hearts post-MI (*n* = 6–8). (d) The relative mRNA levels of hypertrophic markers: ANP, BNP, *β*-MHC, and *α*-MHC in the indicated mice hearts post-MI (*n* = 6). (e) PSR staining and statistical analysis of the heart in the indicated mouse hearts post-MI (left, PSR staining; right, statistical analysis of the LV collagen volume (%), *n* = 25+ fields per experimental group). (f) The relative mRNA levels of fibrosis markers: collagen I and collagen III in the indicated mice hearts post-MI (*n* = 6). ^∗^*p* < 0.05 versus vehicle-sham and ^#^*p* < 0.05 versus vehicle-MI.

At 2 weeks after MI, the ratios of heart weight (HW)/body weight (BW), HW/tibial length (TL), and lung weight (LW)/BW were remarkably increased in vehicle-treated mice (Figure 2(a)) as well as the cross-section area (CSA) of cardiomyocytes (Figure 2(b)). Aucubin treatment inhibited these alterations. Consistently, aucubin also hampered the increases of LVEDd and LVESd post-MI (Figure 2(c)). Additionally, aucubin decreased the transcription of hypertrophic marker level, while increasing the *α*-myosin heavy chain (*α*-MHC) expression level compared with that in the vehicle group (Figure 2(d)).

Meanwhile, aucubin also decreased interstitial fibrosis post-MI compared with that in the vehicle group (Figure 2(e)) as well as the expression of fibrotic markers (Figure 2(f)). These data indicate that the administration of aucubin alleviates cardiac hypertrophy and fibrosis post-MI.

### 3.3. Aucubin Inhibits Inflammation and Apoptosis

**Figure 3:**
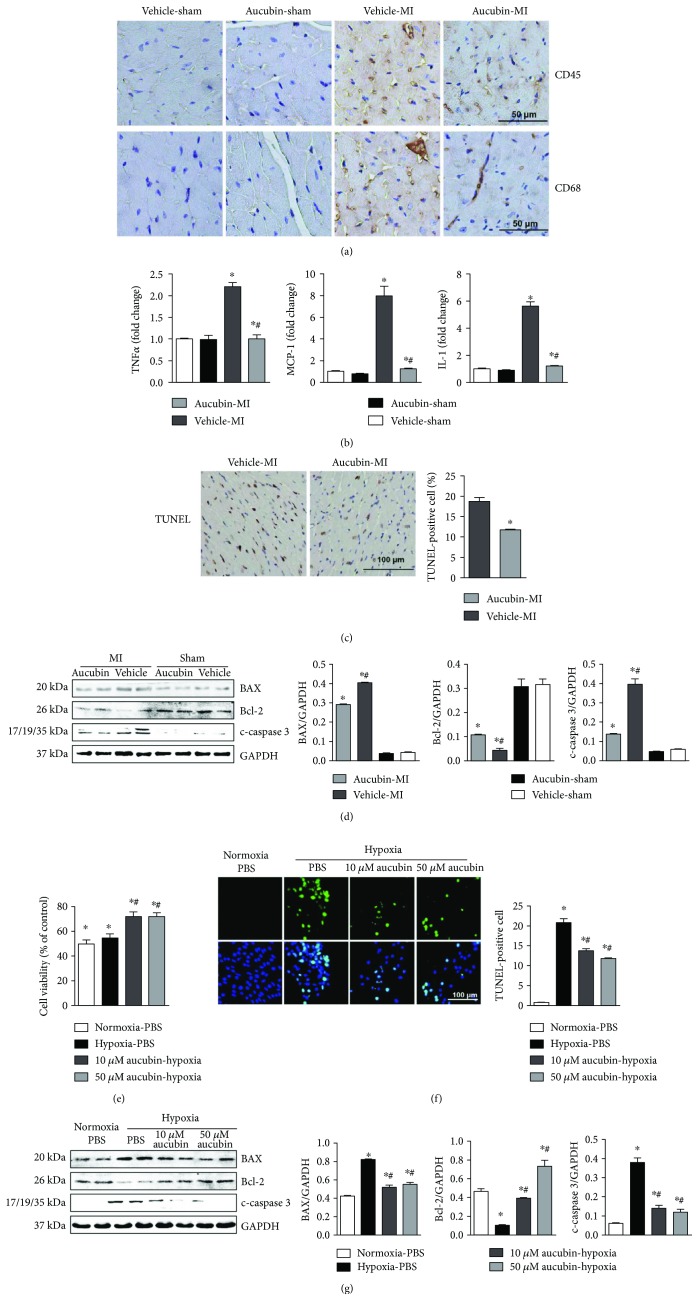
Aucubin inhibits inflammation and apoptosis. (a) Immunohistochemistry staining of CD45- and CD68-positive cells in the heart in the indicated mouse hearts post-MI (*n* = 6). (b) The relative mRNA levels of TNF*α*, MCP-1, and IL-1 in the indicated mouse hearts post-MI (*n* = 6). ^∗^*p* < 0.05 versus vehicle-sham and ^#^*p* < 0.05 versus vehicle-MI. (c) TUNEL staining (left) and quantitation (right) in the indicated mouse hearts post-MI (*n* = 6, ^∗^*p* < 0.05 versus vehicle-MI). (d) Representative Western blots and quantitation of Bax, Bcl-2, and c-caspase 3 in the indicated mouse hearts post-MI (*n* = 6, ^∗^*p* < 0.05 versus vehicle-sham and ^#^*p* < 0.05 versus vehicle-MI). Left, representative blots; right, statistical analysis result. ^∗^*p* < 0.05 versus vehicle-sham and ^#^*p* < 0.05 versus vehicle-MI H9c2 cells were pretreated with aucubin (1 *μ*M, 10 *μ*M, and 50 *μ*M) for 12 h and then exposure to hypoxia for 24 h. (e) Cell counting kit-8 (CCK8) assays were performed to detect cell viability (*n* = 5, ^∗^*p* < 0.05 versus hypoxia and ^#^*p* < 0.05 versus hypoxia + 1 *μ*M aucubin). (f) TUNEL staining (left) and quantitation (right) in the indicated group (*n* = 6). (c) and (g) Western blots of Bax, Bcl-2, and c-caspase 3 in the indicated group (left, representative blots; right, statistical analysis result) (*n* = 4 sample). ^∗^*p* < 0.05 versus normoxia + PBS and ^#^*p* < 0.05 versus hypoxia + PBS. All experiments were repeated independently three times.

Immunohistochemical staining of CD45 and CD68 (the leukocyte and macrophage markers, resp.) in the infarcted border zone revealed a decreased infiltration of leukocytes and macrophages in aucubin-treated mouse hearts compared with the vehicle mouse hearts (Figure 3(a)). Moreover, aucubin decreased the expression level of those proinflammatory cytokines post-MI compared with the vehicle-MI group (Figure 3(b)).

TUNEL assays were used to detect cardiomyocyte apoptosis. MI induced larger numbers of apoptosis in cardiomyocytes (Figure 3(c)) with decreased expression of antiapoptotic protein Bcl-2 and decreased expression of proapoptotic protein Bax and cleaved caspase 3 (Figure 3(d)), while aucubin reduced the apoptotic cell number and increased the Bcl-2 expression and decreased Bax and cleaved caspase 3 expression (Figures 3(c) and 3(d)).

The direct effects of aucubin on cardiomyocytes were determined in vitro study. After pretreated with aucubin (1 *μ*M, 10 *μ*M, and 50 *μ*M) for 12 h, cardiomyocytes were exposed to hypoxia for 24 h. The CKK8 result revealed that both 10 *μ*M and 50 *μ*M aucubin could enhance cell viability, while 1 *μ*M aucubin, it seems, could not protect cardiomyocytes after exposure to hypoxia for 12 h (Figure 3(e)). Thus, 10 *μ*M and 50 *μ*M aucubin were selected to perform the further study. Consistent with our in vivo results, both 10 *μ*M and 50 *μ*M aucubin, respectively, decreased the number of TUNEL-positive cells (Figure 3(f)). Accordingly, the expressions of Bax and cleaved caspase 3 were remarkably reduced, and antiapoptotic protein Bcl-2 was enhanced after treatment with aucubin (Figure 3(g)).

### 3.4. Aucubin Inhibits MI and Hypoxia-Induced Activation of the TNF*α*-ASK1-JNK Signal

**Figure 4:**
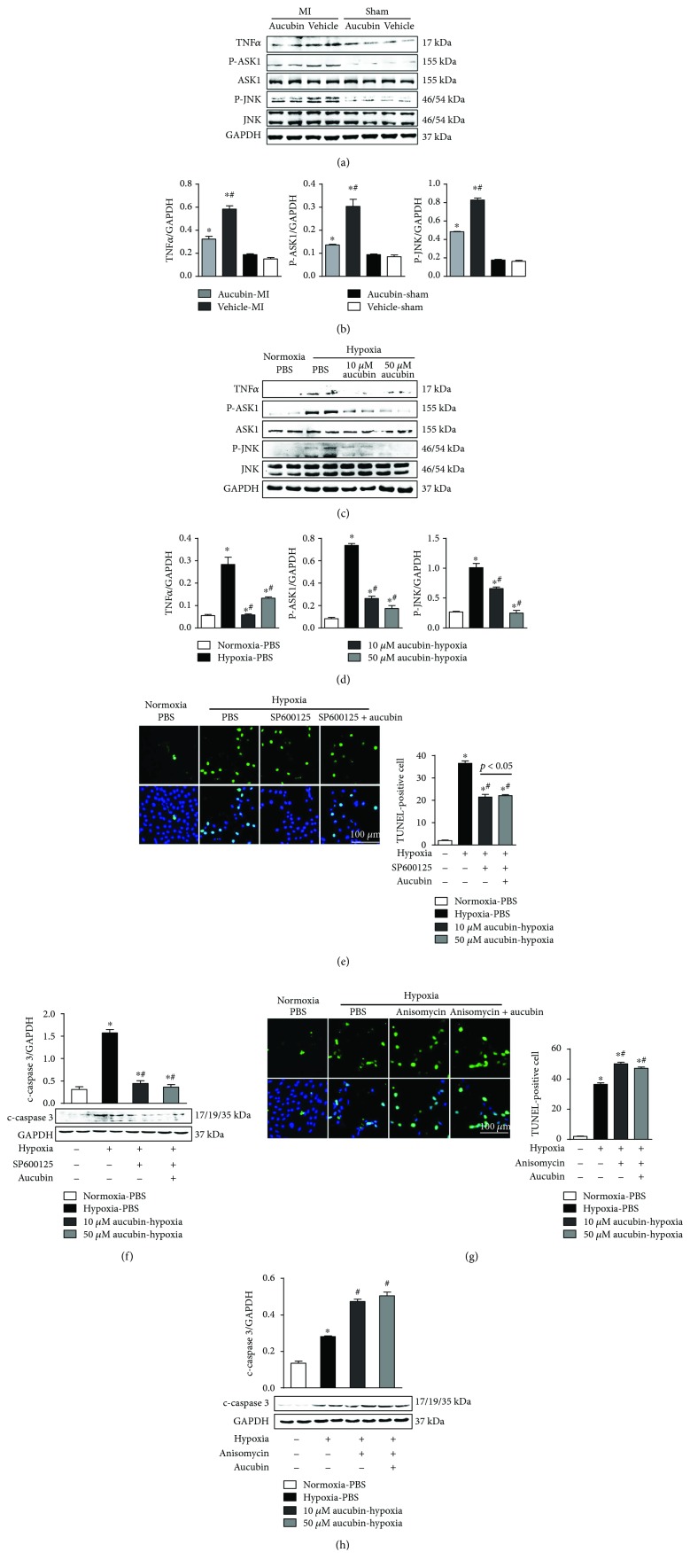
Aucubin inhibits MI and hypoxia-induced activation of the TNF*α*-ASK1-JNK signal. (a) and (b) Western blot analysis of TNF*α*, phosphorylated (p-) and total ASK1, and JNK in the indicated mouse hearts post-MI ((a) representative blots and (b) statistical analysis result) (*n* = 6, ^∗^*p* < 0.05 versus vehicle-sham and ^#^*p* < 0.05 versus vehicle-MI). (c) and (d) Western blot analysis of TNF*α*, phosphorylated (p-) and total ASK1, and JNK in the aucubin- (10 *μ*M, 50 *μ*M) pretreated H9c2 cardiomyocytes after exposure to hypoxia for 24 h ((c) representative blots and (d) statistical analysis result) (*n* = 4 sample). ^∗^*p* < 0.05 versus normoxia + PBS and ^#^*p* < 0.05 versus hypoxia + PBS (e–h). H9c2 cardiomyocytes were pretreated with a JNK inhibitor, SP600125 (10 *μ*M), or JNK agonist, anisomycin (40 ng/ml), and/or aucubin (50 *μ*M) for 12 h and then exposed to hypoxia for 24 h. (e) and (g) TUNEL staining and quantitation in the indicated group (*n* = 6 sample). (f) and (h) Representative Western blots and quantitation of c-caspase 3 in the indicated group (*n* = 4 sample). ^∗^*p* < 0.05 versus normoxia + PBS and ^#^*p* < 0.05 versus hypoxia + PBS. All experiments were repeated independently three times.

To identify the underlying mechanism of aucubin administration, we detected the signaling pathways involved in cardiac remodeling post-MI. MI induced increased expression of TNF*α* and increased phosphorylated levels of JNK1/2 and ASK1. These levels were totally blocked by aucubin treatment (Figures 4(a) and 4(b)). Consistently, the hypoxia-induced enhanced expression of TNF*α* and activation of ASK1 and JNK1/2 were inhibited by 50 *μ*M aucubin pretreatment (Figures 4(c) and 4(d)).

To further verify whether aucubin targets on TNF*α*-ASK1-JNK signaling cascades to perform its cardioprotective effects, JNK inhibitor (SP600125) and agonist (anisomycin) were used. SP600125 significantly reduced hypoxia-induced apoptosis. Importantly, aucubin could not further improve the reduced cell apoptosis by SP600125 (Figures 4(e) and 4(f)). JNK agonist, anisomycin, accelerated hypoxia-induced apoptosis while abolishing the antiapoptotic effect of aucubin (Figures 4(g) and 4(h)). Taken together, inhibition of the TNF*α*-ASK1-JNK signaling pathway accounted for the protective role of aucubin on cardiac remodeling.

### 3.5. Aucubin Attenuates Oxidative Stress and Increases Thioredoxin (Trx) In Vivo and In Vitro

**Figure 5:**
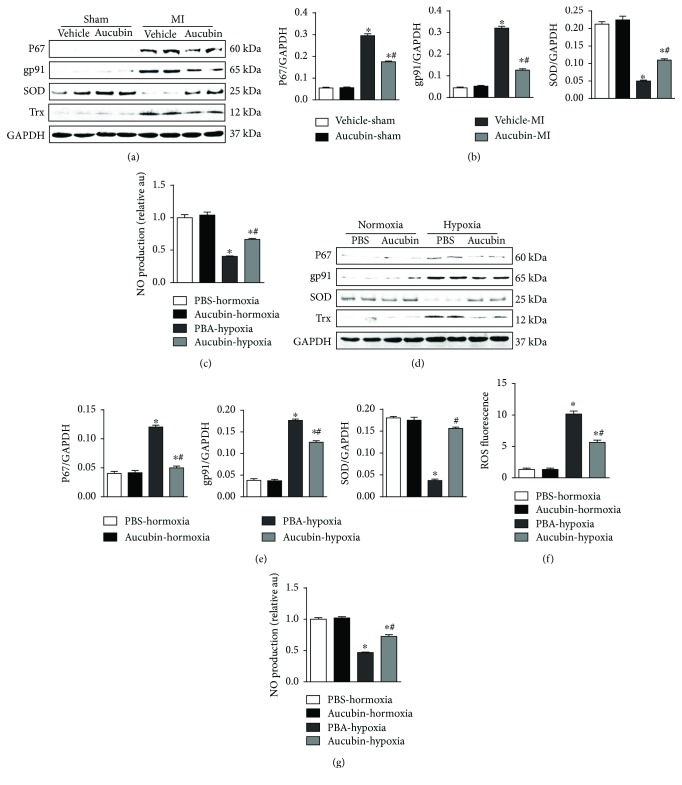
Aucubin attenuates oxidative stress and increases thioredoxin (Trx) in vivo and vitro. (a) and (b) Western blot analysis of P67, gp91, SOD, and Trx in the indicated heart tissue post-MI ((a) representative blots and (b) statistical analysis result) (*n* = 6). (c) NO production in the indicated heart tissue. ^∗^*p* < 0.05 versus vehicle-sham and ^#^*p* < 0.05 versus vehicle-MI. (d) and (e) Western blot analysis of P67, gp91, SOD, and Trx in the aucubin- (50 *μ*M) pretreated H9c2 cardiomyocytes after exposure to hypoxia for 24 h ((a) representative blots and (e) statistical analysis result) (*n* = 4 sample). (f) ROS level detected by DCF-DA in the indicated group (*n* = 6 sample). (g) NO production in the indicated group (*n* = 6 sample). ^∗^*p* < 0.05 versus normoxia + PBS and ^#^*p* < 0.05 versus hypoxia + PBS. All experiments were repeated independently three times.

Under physiological conditions, Trx bonds with ASK1 leading to its inactivation. Under various stimulations, ASK1 is phosphorylated and dissociated from Trx, leading to its activation [17]. We then detected the oxidative stress after aucubin treatment. The NADPH oxidase subunits, P67 and gp91, were decreased by aucubin, while antioxidants SOD and Trx were increased by aucubin treatment in vivo remodeling both mouse heart and cardiomyocytes exposed to hypoxia (Figures 5(a), 5(b), 5(d), and 5(e)). Aucubin also decreased ROS production in cardiomyocytes exposed to hypoxia (Figure 5(f)). We further found that nitric oxide (NO) production decreased in remodeling mouse heart and hypoxia-damaged cardiomyocytes and increased after aucubin treatment (Figures 5(c) and 5(g)).

### 3.6. nNOS Mediates the Protective Effects of Aucubin In Vitro

**Figure 6:**
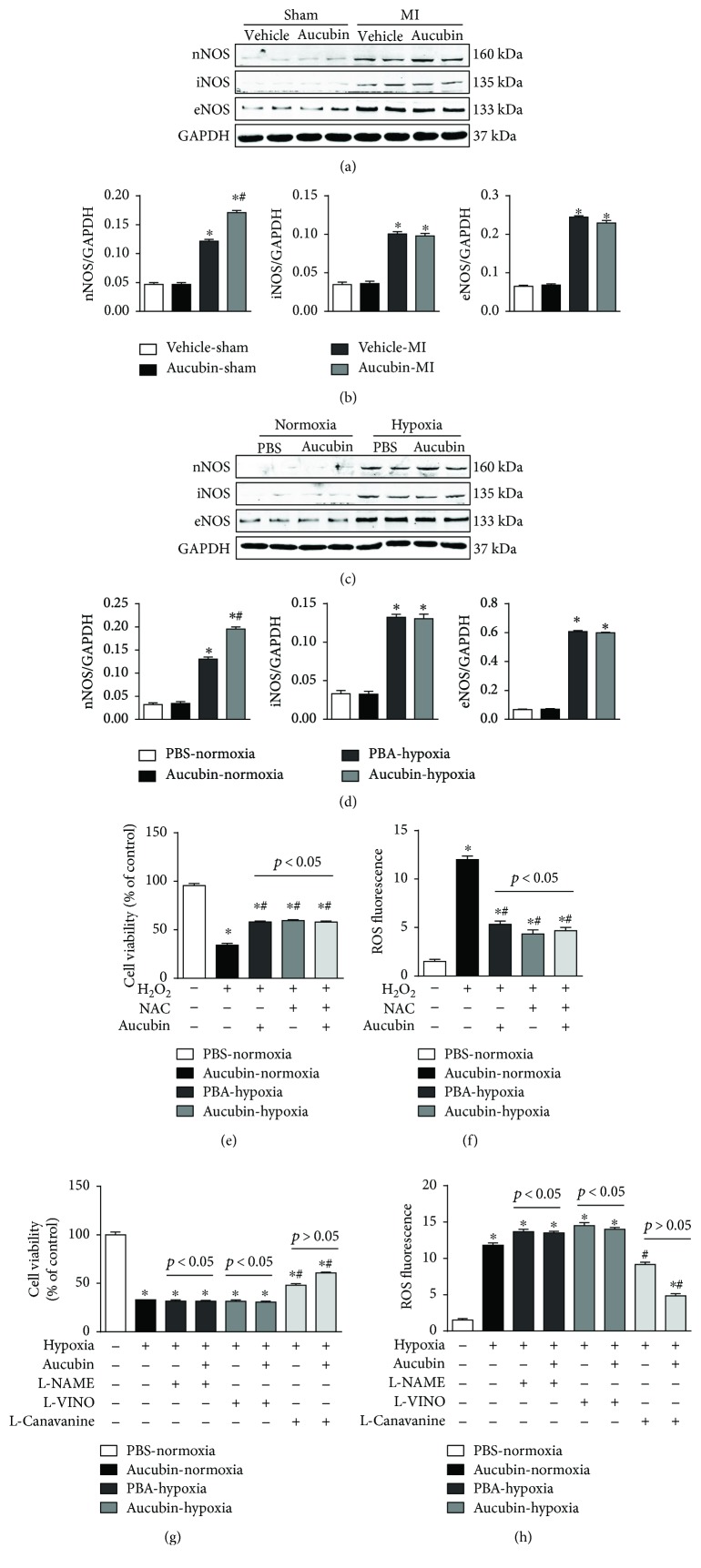
nNOS mediates the protective effects of aucubin in vitro. (a) and (b) Western blot analysis of nNOS, iNOS, and eNOS in the indicated heart tissue post-MI surgery ((a) representative blots and (b) statistical analysis result) (*n* = 6, ^∗^*p* < 0.05 versus vehicle-sham and ^#^*p* < 0.05 versus vehicle-MI). (c) and (d) Western blot analysis of nNOS, iNOS, and eNOS in the aucubin- (50 *μ*M) pretreated H9c2 cardiomyocytes after exposure to hypoxia for 24 h ((c) representative blots and (d) statistical analysis result) (*n* = 4 sample). ^∗^*p* < 0.05 versus normoxia + PBS and ^#^*p* < 0.05 versus hypoxia + PBS. (e) and (f) Cardiomyocytes were pretreated with NAC or aucubin and then stimulated with H_2_O_2_. (f) Cell viability detected by CKK8 assay in the indicated group (*n* = 6 samples). (f) ROS level was detected by DCF-DA in the indicated group (*n* = 6 samples). (g) and (h) Cardiomyocytes were pretreated with L-NAME, L-VINO, or L-canavanine or/and aucubin and then exposed to hypoxia. (g) Cell viability detected by CKK8 assay in the indicated group (*n* = 6 samples). (h). ROS level was detected by DCF-DA in the indicated group (*n* = 6 samples). ^∗^*p* < 0.05 versus normoxia + PBS and ^#^*p* < 0.05 versus hypoxia + PBS. All experiments were repeated independently three times.

Nitric oxide synthases (NOSs) are responsible for the synthesis of NO from L-arginine. Thus, NOS expression was detected after aucubin treatment. Neuronal NOS (nNOS), inducible NOS (iNOS), and endothelial NOS (eNOS) were all upregulated in both remodeled mouse heart and hypoxia-damaged cardiomyocytes. However, only nNOS was further increased by aucubin treatment (Figures 6(a) and 6(c)). We further confirm the role of nNOS on aucubin-mediated protective effects. Cardiomyocytes were pretreated with aucubin and ROS scavenger N-acetyl-cysteine (NAC) and then stimulated with H_2_O_2_ to induce oxidative stress. H_2_O_2_ decreased the cell viability and increased ROS production, while both aucubin and NAC could preserve the cell viability and decrease the level of ROS. Synergism of aucubin and NAC could not enhance these protective effects (Figures 6(e) and 6(f)). Cardiomyocytes were pretreated with the nonselective NOS inhibitor (L-NAME), selective nNOS inhibitor (L-VINO), or selective iNOS inhibitor (L-canavanine), and aucubin was then exposed to hypoxia. Both L-NAME and L-VINO reversed the protective effects of aucubin, as assessed by decreased cell viability and increased ROS level (Figures 6(g) and 6(h)). Additionally, the iNOS inhibitor could not block the protective effects of aucubin, as evidenced by increased cell viability and decreased ROS level (Figures 6(g) and 6(h)).

### 3.7. nNOS Knockout (KO) Abolished the Antiremodeling Effects of Aucubin In Vivo

**Figure 7:**
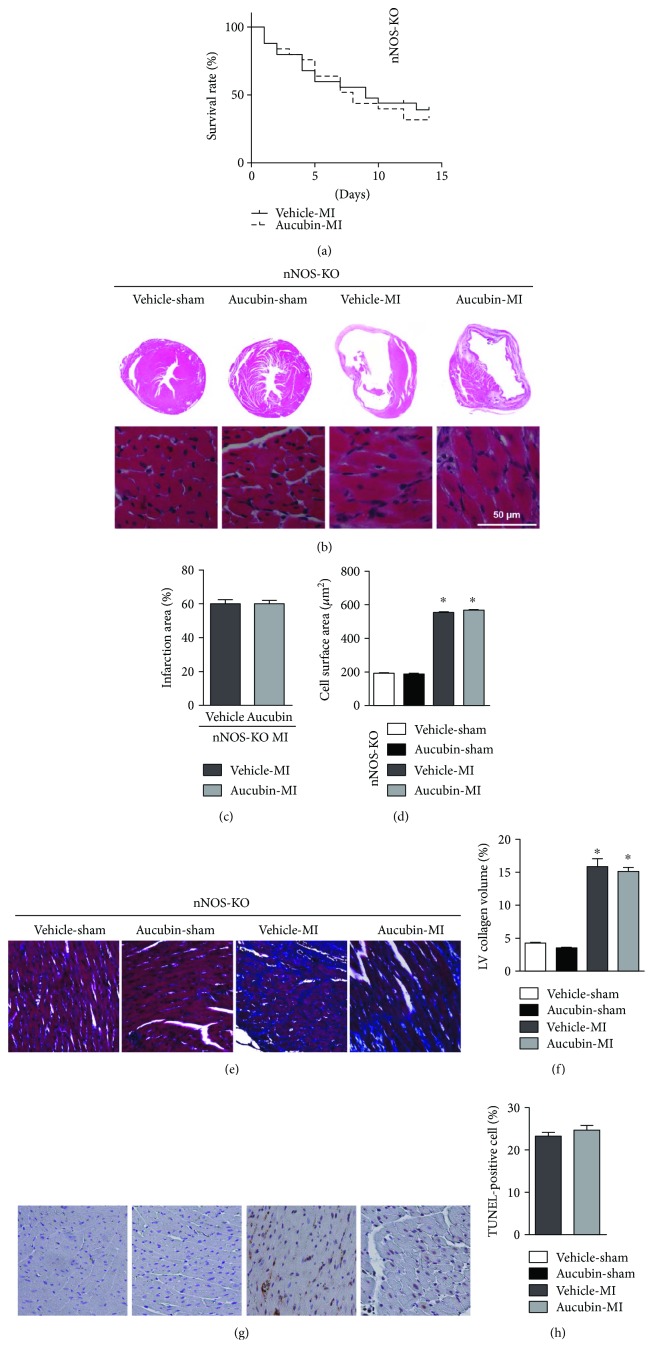
nNOS-KO abolished the antiremodeling effects of aucubin in vivo. (a) Kaplan-Meier survival analysis of nNOS-KO mice in vehicle-MI and aucubin-MI group during 2 weeks after MI. (b–d) H&E staining ((b) *n* = 6) and statistical analysis of the infarction area (c) and cross-sectional area ((d) CSA, *n* = 100+ cells per experimental group). (e) and (f) PSR staining of the heart in the indicated groups [(e) PSR staining (*n* = 6) and (f) statistical analysis of the LV collagen volume (%), *n* = 25+ fields per experimental group]. (g) and (h) TUNEL staining (g) (*n* = 6) and quantitation (h) in the hearts of vehicle and aucubin-treated nNOS-KO mice at 2 weeks post-MI. ^∗^*P* < 0.05 vs. the Vehicle-sham group.

nNOS KO mice were subjected to LAD surgery and administered to aucubin treatment. Two weeks after MI, nNOS-KO mice experienced a high death rate (Figure 7(a)), augmented infarction area (Figures 7(b) and 7(c)), increased cardiac hypertrophy (Figures 7(b) and 7(d)), fibrosis (Figures 7(e) and 7(f)), and cell apoptosis (Figures 7(g) and 7(h)). However, aucubin treatment could not improve these deteriorated outcomes (Figures 7(a) and 7(h)).

## 4. Discussion

Myocardial ischemia-mediated necrosis and apoptosis after MI promote the progression of heart failure [18]. Thus, targeting apoptosis is a promising strategy for preventing cardiac remodeling. Studies have reported the antiapoptotic effect of aucubin in PC12 cells [19] and neuronal cells [20]. In this study, we found that the MI-induced cell apoptosis and hypoxia-induced cardiomyocyte apoptosis were both attenuated by aucubin treatment, which protected the heart from the subsequent inflammatory and hypertrophic response and fibrosis, ultimately leading to improved cardiac remodeling and increased survival rate in mice. Two pathways mediate the apoptosis in the infarcted heart: one is the intrinsic pathway activated by mitochondrial signaling; the other is the extrinsic pathway mediated by the binding of death ligands to death receptors on the cell surface [18]. Studies have revealed that aucubin is involved in both intrinsic [20] and extrinsic pathways [21]. Our data suggested that aucubin regulated the expression of Bcl-2 family proteins and decreased cleavage of caspase 3, which indicates inhibition of the intrinsic pathway.

TNF*α*, released by many inflammatory cells as well as cardiomyocytes after MI, is known to mediate apoptosis [1]. TNF*α* binds to the TNF*α* receptor leading to the activation of ASK1 [16]. After activation, ASK1 activates JNK by recruiting to oligomerized IRE1 complexes and leads its activation [17, 22]. The activated JNK can phosphorylate proapoptotic protein, Bim, and block antiapoptotic protein, Bcl-2, leading to the imbalance of the proapoptotic protein/antiapoptotic protein ratio and consequently cell apoptosis [17]. In our study, TNF*α* was found to increase post-MI and hypoxia exposure, which induced activation of the ASK1/JNK pathway. This signaling was blocked by aucubin treatment. Interestingly, the JNK inhibitor reverses the protective effects of aucubin on cardiomyocytes in a hypoxia model. These data suggest that aucubin exerts anticardiac remodeling effects via inhibition of the ASK1/JNK pathway.

ASK1 acts as a redox sensor. Under physiological conditions, Trx bonds with ASK1 leading to its inactivation. Under various stimulations (TNF*α* or various oxidants), ASK1 is phosphorylated and dissociated from Trx, leading to its activation [23]. The activation of ASK1/JNK signaling by oxidative stress and TNF*α* leads to apoptosis in various cell types. Thereby, as a negative regulator, Trx is a potential target [24]. We found that aucubin attenuation exceeded oxidative stress and preserved Trx in remodeled mouse heart and cardiomyocytes and thus blocked the activation of ASK1. NO, a free-radical gas, plays an important role in redox signal in heart diseases. In the heart, NO is mainly derived from the classic L-arginine-NOS-NO pathway [25]. NO can target various types of proteins, leading to an array of downstream signaling cascade activations. Of interest, nNOS expressed in all parts of the heart, and its derived NO plays a fundamental role in the heart's pathophysiological process [26]. nNOS shuttles into the nucleus, banding and regulating the elements involved in oxidative phosphorylation and mitochondrial biogenesis [26]. Moreover, by directly targeting heart oxidases, such as Trx, oxidoreductase, and NADPH oxidase, nNOS-derived NO controls redox signal and the downstream effects in the heart [27]. We found that aucubin-triggered reduction in oxidative stress was mediated by enhancement of the nNOS-NO pathway. Aucubin increased nNOS expression leading to the augmented NO production and the subsequent diminished oxidative stress. This protection of aucubin was totally abolished by nNOS deficiency.

In conclusion, our data evidence that aucubin relieves the cardiac remodeling process in response to MI and improves cardiac function and survival rate in mice. These protective effects of aucubin involve the regulating of the nNOS/NO pathway, which leads to the diminished oxidative stress and subsequent blocking of ASK1/JNK signaling. Some limitations existed in our study. First, only one dosage was used in our in vivo study. Future study about the distribution of aucubin in blood after intraperitoneal injection and the proper dosage should be determined. Second, whether aucubin could influence other cell types and signaling pathways in the process of cardiac remodeling needs further study.
